# Identifying Effective Connectivity between Stochastic Neurons with Variable-Length Memory Using a Transfer Entropy Rate Estimator

**DOI:** 10.3390/brainsci14050442

**Published:** 2024-04-29

**Authors:** João V. R. Izzi, Ricardo F. Ferreira, Victor A. Girardi, Rodrigo F. O. Pena

**Affiliations:** 1 Department of Statistics, Federal University of São Carlos, São Carlos 13565-905, SP, Brazil; 2 Department of Biological Sciences, Florida Atlantic University, Jupiter, FL 33458, USA; 3 Stiles-Nicholson Brain Institute, Florida Atlantic University, Jupiter, FL 33458, USA

**Keywords:** effective connectivity, transfer entropy, conditional independence, causality, hypothesis testing, interacting variable-length Markov chains

## Abstract

Information theory explains how systems encode and transmit information. This article examines the neuronal system, which processes information via neurons that react to stimuli and transmit electrical signals. Specifically, we focus on transfer entropy to measure the flow of information between sequences and explore its use in determining effective neuronal connectivity. We analyze the causal relationships between two discrete time series, $X:=\left\{X_{t}:t\in Z\right\}$ and $Y:=\left\{Y_{t}:t\in Z\right\}$, which take values in binary alphabets. When the bivariate process (X,Y) is a jointly stationary ergodic variable-length Markov chain with memory no larger than *k*, we demonstrate that the null hypothesis of the test—no causal influence—requires a zero transfer entropy rate. The plug-in estimator for this function is identified with the test statistic of the log-likelihood ratios. Since under the null hypothesis, this estimator follows an asymptotic chi-squared distribution, it facilitates the calculation of *p*-values when applied to empirical data. The efficacy of the hypothesis test is illustrated with data simulated from a neuronal network model, characterized by stochastic neurons with variable-length memory. The test results identify biologically relevant information, validating the underlying theory and highlighting the applicability of the method in understanding effective connectivity between neurons.

## 1. Introduction

Estimating the effective connectivity between neurons in the brain is not an easy task [1,2,3,4,5]. There are many ways to unveil causal relationships in their multiple scales, such as from neurons to brain regions. Experiments with external stimuli are commonly used for this inference process, where the spikes and the activity of a neuron are related to a second neuron that is connected to the first one if the perturbation allows us to see that [6]. These procedures focus on an improvement in the prediction of the future activity of the second neuron (the receiver) by incorporating information produced by the past activity of the first neuron (the sender of the perturbation), which is seen as a causal interaction between these neurons [7].

Admittedly, connectivity estimation is not straightforward due to the noisy nature of neuronal signals. Recordings of electrophysiological patterns in vitro and in vivo reveal that the neuronal activity is highly irregular and difficult to predict [8,9,10]. Intrinsic variability is apparent in the response of neurons, even to frozen stimulation [11,12]. Experimental data suggest that neurons, synapses, and the network system operate in an inherently stochastic framework [13,14,15]. Accordingly, the mathematical description of neuronal phenomena can be treated in probabilistic terms, i.e., describing the process of spiking as a stochastic process.

Determining which stochastic process is more suitable is a matter of debate. It is, however, reasonable to consider that the probability of a neuron spiking is conditioned by the knowledge of its past temporal response. Hence, this probability is greater the further in the past the last spike of the neuron in question is. This implies that the stochastic process that models the activity of this neuron is not Markovian with full memory, as shown by some works in the literature [16,17,18]. The activity of a neuron could, therefore, be reasonably modeled by a stochastic process whose dependence on the past is variable in scope.

The class of Markov chains with variable-length memory became popular in the statistical and probabilistic community with the work by [19]. The processes in this class are still Markovian of fixed order but with transition probabilities that do not depend on a fixed number of past states, taking into account, on the other hand, the dependency structure present in the data. These relevant sequences of past states are called contexts, and the set of contexts can be represented as a rooted tree, namely a context tree. When considering a variable-length memory, we have more informative models that are flexible and parsimonious compared to Markov chains with full memory.

Given a trajectory of the Markov chain with a variable-length memory, we can estimate transition probabilities using, for example, a plug-in estimator. A way of estimating connectivity and disambiguate spurious correlations from actual connections is by inferring the information that flows from one neuronal spike train to another. For this, we can use information-theoretical measures [20,21,22], which are functions of these transition probabilities. Thus, the estimation of the transition probabilities is essential. In this work, we consider the modeling of the neuronal spike trains by way of Markov chains with variable-length memory and use transfer entropy to understand the transmission of information between neurons over a finite time interval.

Transfer entropy (TE), an information-theoretic measure for quantifying time-directed information transfer between joint processes, was proposed by [20] and independently by [23] as an effective measure of causality. A closely related concept that measures information transport is the transfer entropy rate (TER). These measures can quantify the strength and direction of coupling between simultaneously observed systems [24,25]. Consequently, TE and TER are widely used in neuroscience today to assess connectivity from neuronal datasets [3,26,27,28,29,30,31,32,33]. In this sense, these measures allow us to study both linear and nonlinear causality relations between neuronal spike trains, described as discrete random processes. In this article, we are interested in the application of these measures to the detection of effective neuronal connectivity between neurons with variable-length memory. In other words, we aim to test for the absence of causal influence between neurons. Under fair conditions, the null hypothesis, which corresponds to the absence of causal influence, is equivalent to the requirement that the transfer entropy rate is equal to zero [34].

To test the statistical significance of a connectivity value and determine whether connectivity is detected, we use the plug-in estimator for the transfer entropy rate, which is identified with the log-likelihood ratio test statistic for the desired test. According to [34,35], this statistic is asymptotically $\chi^{2}$ distributed under the null hypothesis, facilitating the computation of *p*-values when used on simulated data. In this work, the test is employed in the analysis of spike trains simulated from a space-time framework inspired by the Galves and Löcherbach model [36], which is built on the simple and biologically plausible assumption that the membrane potential of each neuron is reset every time it spikes. The authors construct a stationary version of the process using probabilistic tools and obtain an explicit upper bound for the correlation between successive inter-spike intervals. This enables the application of the proposed statistical test to samples generated from this model. The effectiveness of the resulting hypothesis test is illustrated in these simulated data, which identifies interesting and biologically relevant information.

The problem of testing effective connectivity between neurons based on transfer entropy has been considered in the literature using surrogate data [3,37]. In general, generating surrogate data with the same properties as the original data but without dependencies between signals is difficult. In this sense, feasibility emerges when collecting sufficiently large samples, and when the test statistic has a known asymptotic distribution, the use of parametric tests is a good alternative. Recently, parametric tests have been used to detect connectivity between neurons using a test statistic based on the plug-in estimator of directed information, assuming that the bivariate process is Markovian with full memory [38,39]. To the best of our knowledge, this is the first time that testing for causality using a transfer entropy has been performed in the more general scenario of Markov chains with variable-length memory, based on a transfer entropy rate plug-in estimator. Thus, this work complements existing studies on transfer entropy estimation and effective connectivity detection between neurons.

The remainder of this article is organized as follows. In the next section, we establish our notations and review preliminary definitions and concepts, particularly those concerning the neuronal network model, transfer entropy, and the estimation of the transfer entropy rate. Section 3 introduces the hypothesis test we use to detect causal influence between stochastic neurons. In Section 4, we apply transfer entropy to the identification of effective connectivity between a pair of stochastic neurons using synthetic data generated from the random network model described in Section 2. Lastly, we end this article with our conclusions in Section 5.

## 2. Notations, Definitions, and Preliminary Notions

In this paper, we denote random variables in uppercase letters, stochastic chains in uppercase bold letters, and the specific values assumed by them in lowercase letters. Calligraphic letters denote the alphabets where random variables take values. Subscripts denote the outcome’s position in a sequence, for example, $X_{t}$ generally indicates the $t^{th}$ outcome of the process X. For any integers *j* and *k* such that j≤k, we use the notation $x_{j}^{k}$ for finite sequences $\left(x_{j},\ldots ,x_{k}\right)$, $x_{-\infty }^{k}$ for left-infinite sequences $\left(\ldots ,x_{k-1},x_{k}\right)$, and $x_{k}^{+\infty }$ for right-infinite sequences $\left(x_{k},x_{k+1},\ldots \right)$. We use the convention that if j>k, $x_{j}^{k}$ is the empty sequence. We use analogous notations for sequences of random variables.

### 2.1. Neuronal Spike Trains as Stochastic Processes

Throughout this paper, we assume that we record the neuronal activity over a finite time horizon. The sequence of times at which an individual neuron in the nervous system generates an action potential is termed a *spike train*. It is useful to consider the times of spike occurrence with a certain degree of accuracy, which is called the *bin size* [40]. In this sense, the bin size refers to the duration of time over which neural activity is aggregated or binned for analysis. For a small enough bin size (10 ms is a typical choice), the spike train may be represented as a binary sequence $x_{1}^{n}\in {\left\{0,1\right\}}^{n}$, where

$$x_{t}=\left\{\begin{array}{cc} 1, & \;\mathrm{if}\;\mathrm{the}\;\mathrm{neuron}\;\mathrm{spikes}\;\mathrm{at}\;\mathrm{the}\;t^{th}\;\mathrm{bin},\\ 0, & \;\mathrm{otherwise}, \end{array}\right.$$

for every t=1,2,…,n. The appropriate bin size to use depends on the specific experimental design and the characteristics of the data being analyzed. In general, the bin size is chosen to strike a balance between capturing relevant details of the neuronal activity and having sufficient statistical power. This typically involves selecting a bin size that is small enough to capture important features of the data but not so small that the resulting spike counts are noisy or unreliable.

Recordings of neuronal activity reveal irregular spontaneous activity of neurons and variability in their response to the same stimulus [41,42,43,44,45]. Thus, the experimental data suggest that spike trains should be modeled from a probabilistic point of view. In this context, and to give a probability measure to describe the process of spiking as a sequential process, we assume that the activity of a neuron is described by a discrete-time homogeneous stochastic chain $X:=\{X_{t}:t\in Z\}$ defined on a suitable probability space (Ω,F,P), where

$$X_{t}=\left\{\begin{array}{cc} 1, & \;\mathrm{if}\;\mathrm{the}\;\mathrm{neuron}\;\mathrm{spikes}\;\mathrm{at}\;\mathrm{the}\;t^{th}\;\mathrm{bin},\\ 0, & \;\mathrm{otherwise}, \end{array}\right.$$

for every t∈Z.

In this paper, we assume that the sample spike train is generated by a stochastic source. This means that at each bin, conditional on the whole past, there is a fixed probability of obtaining a spike. Neurons exhibiting this characteristic are arranged in such a way that they share similar biophysical properties and are collectively referred to as *stochastic neurons*.

The randomness introduced by stochastic neurons can be useful in training neural network models because it can help prevent overfitting and improve the network’s ability to generalize to new data. In this work, we are interested in detecting the effective connectivity between a pair of stochastic neurons using synthetic data generated from such random network models.

### 2.2. Neuronal Network Model

Let *I* be a finite set of neurons, and assume that the bins are indexed by the set Z. In this context, the network of neurons is described by a discrete-time homogeneous stochastic chain $X:=\left\{X_{t}^{\left(i\right)}:i\in I,t\in Z\right\}$. For each neuron i∈I at each bin t∈Z,

$$X_{t}^{\left(i\right)}=\left\{\begin{array}{cc} 1, & \;\mathrm{if}\;\mathrm{neuron}\;i\;\mathrm{spikes}\;\mathrm{at}\;\mathrm{the}\;t^{th}\;\mathrm{bin},\\ 0, & \;\mathrm{otherwise}. \end{array}\right.$$

Moreover, whenever we say time t∈Z, it should be interpreted as time bin *t*. For notational convenience, we write the configuration of X at time t∈Z by $X_{t}:=\left\{X_{t}^{\left(i\right)}:i\in I\right\}$ and the path of X associated with neuron i∈I as $X^{\left(i\right)}:=\left\{X_{t}^{\left(i\right)}:t\in Z\right\}$. We use analogous notation for the observed configuration of X at time t∈Z and the observed path of X associated with a neuron i∈I.

In what follows, *P* denotes the law of the neuronal network X. In this network, the stochastic chain X has the following dynamic. At each time step, conditional on the whole past, neurons update independently from each other, i.e., for any t∈Z and any choice $x_{t}^{\left(i\right)}\in \left\{0,1\right\}$, i∈I, we have

(1) $$P\left(\left.\underset{i\in I}{⋂}\left\{X_{t}^{\left(i\right)}=x_{t}^{\left(i\right)}\right\}\right|X_{-\infty }^{t-1}=x_{-\infty }^{t-1}\right)=\prod_{i\in I}P\left(\left.X_{t}^{\left(i\right)}=x_{t}^{\left(i\right)}\right|X_{-\infty }^{t-1}=x_{-\infty }^{t-1}\right),$$

where $x_{-\infty }^{t-1}$ is a left-infinite configuration of X.

Moreover, the probability that neuron i∈I spikes at bin t∈Z, conditional on the whole past, is an increasing function of its membrane potential. In other words, for each neuron i∈I at any t∈Z,

(2) $$P\left(\left.X_{t}^{\left(i\right)}=1\right|X_{-\infty }^{t-1}=x_{-\infty }^{t-1}\right)=\phi \left(v_{t-1}^{\left(i\right)}\right),$$

where $v_{t}^{\left(i\right)}\in R$ denotes the membrane potential of neuron i∈I at time t∈Z and ϕ:R→[0,1] is an increasing function called the *spiking rate function*.

The membrane potential of a given neuron i∈I is affected by the actions of all other neurons interacting with it. More precisely, the membrane potential of a given neuron i∈I depends on the influence received from its presynaptic neurons since its last spiking time. In this sense, the probability of neuron i∈I spiking increases monotonically with its membrane potential. Whenever neuron i∈I fires, its membrane potential is reset to a resting value, and at the same time, postsynaptic current pulses are generated, modifying the membrane potential of all its postsynaptic neurons. When a presynaptic neuron j∈I−{i} fires, the membrane potential of neuron i∈I changes. The contribution of neuron j∈I to the membrane potential of neuron i∈I is either excitatory or inhibitory, depending on the sign of the synaptic weight of neuron *j* on neuron *i*. Moreover, the membrane potential of each neuron in the network is affected by the presence of leakage channels in its membrane, which tends to push its membrane potential toward the resting potential. This spontaneous activity of neurons is observed in biological neuronal networks.

Assuming the above description, we may consider stochastic neurons with several kinds of short-term memory. In this article, we explore a stochastic neuron model inspired by the GL model [36], where neuronal spike trains are prescribed by interacting chains with variable-length memory.

For each neuron i∈I at any bin t∈Z, we can write

$$v_{t-1}^{\left(i\right)}=\left\{\begin{array}{cc} 0, & \;\mathrm{if}\;x_{t-1}^{\left(i\right)}=1,\\ \beta_{i}+\sum_{j\in I}\omega_{j\to i}\sum_{s=L_{t}^{\left(i\right)}+1}^{t-1}\frac{x_{s}^{\left(j\right)}}{2^{t-L_{t}^{\left(i\right)}-1}}, & \;\mathrm{otherwise}, \end{array}\right.$$

where $\omega_{j\to i}\in R$ is the synaptic weight of neuron *j* on neuron *i*, $\beta_{i}\in R$ is the spontaneous activity of neuron *i*, and $L_{t}^{\left(i\right)}$ is the last spike time of neuron i∈I before time t∈Z, i.e.,

$$L_{t}^{\left(i\right)}:=\sup\left\{s<t:x_{s}^{\left(i\right)}=1\right\},\;\forall i\in I.$$

Therefore, for each neuron i∈I at any t∈Z, we may rewrite (2) in the following way

(3) $$P\left(\left.X_{t}^{\left(i\right)}=1\right|X_{-\infty }^{t-1}=x_{-\infty }^{t-1}\right)=\phi \left(\left(1-x_{t-1}^{\left(i\right)}\right)\left(\beta_{i}+\sum_{j\in I}\omega_{j\to i}\sum_{s=L_{t}^{\left(i\right)}+1}^{t-1}\frac{x_{s}^{\left(j\right)}}{2^{t-L_{t}^{\left(i\right)}-1}}\right)\right).$$

Observe that the spiking probability of a given neuron depends on the accumulated activity of the system after its last spike time. Here, we adopt the convention that $L_{t}^{\left(i\right)}\geq t-K$, where *K* is a positive integer number that represents the largest memory length of all stochastic neurons considered in the network. This implies that the time evolution of each single neuron looks like a Markov chain with variable-length memory. This structure of variable-length memory is more appropriate from the estimation point of view because it implies that some transition probabilities of the Markov chain with order *K* are lumped together.

One can show the existence and uniqueness of a stationary stochastic chain X satisfying (1) whose dynamics are given by (3). We refer the interested reader to [36] for a rigorous proof of this result in the GL neuron model.

### 2.3. Transfer Entropy

In this work, we use transfer entropy to assess connectivity from neuronal datasets. This measure allows us to study causality relations between neuronal spike trains described as discrete random processes. Transfer entropy is a statistical tool used to quantify the directed flow of information between different neurons. Specifically, it measures how much information from one signal helps predict the future of another signal, after accounting for the past of both signals.

Let $\left(X,Y\right):=\left\{(X_{t},Y_{t}):t\in Z\right\}$ be a discrete-time jointly homogeneous stochastic chain taking values on the alphabet ${\left\{0,1\right\}}^{2}$ with distribution P∈M, where M is the set of Borelian probability measures defined on the usual sigma-algebra generated by cylinders of ${\left\{0,1\right\}}^{Z}\times {\left\{0,1\right\}}^{Z}$. For any positive integer *k*, the *k*-block transfer entropy from $X:=\left\{X_{t}:t\in Z\right\}$ to $Y:=\left\{Y_{t}:t\in Z\right\}$ is defined as

$$T\left(X_{1}^{k}\to Y_{1}^{k}\right):=H\left(Y_{k}|Y_{1}^{k-1}\right)-H\left(Y_{k}|X_{1}^{k-1},Y_{1}^{k-1}\right),$$

where $H(Y_{k}|Y_{1}^{k-1})$ is the conditional *k*-block entropy of Y, which is given by

$$H\left(Y_{k}|Y_{1}^{k-1}\right):=-\sum_{b_{1}^{k}}P\left(Y_{1}^{k}=b_{1}^{k}\right)\log P\left(Y_{k}=b_{k}\left|Y_{1}^{k-1}=b_{1}^{k-1}\right.\right)$$

and $H\left(Y_{k}|X_{1}^{k-1},Y_{1}^{k-1}\right)$ is the causally conditional *k*-block entropy of X on Y, defined as

$$\begin{array}{cc} H\left(Y_{k}|X_{1}^{k-1},Y_{1}^{k-1}\right) & :=-\sum_{b_{1}^{k}}\sum_{a_{1}^{k-1}}P\left(X_{1}^{k}=a_{1}^{k-1},Y_{1}^{k}=b_{1}^{k}\right)\log\frac{P\left(X_{1}^{k-1}=a_{1}^{k-1},Y_{1}^{k}=b_{1}^{k}\right)}{P\left(X_{1}^{k-1}=a_{1}^{k-1},Y_{1}^{k-1}=b_{1}^{k-1}\right)}\\ \; & =-\sum_{b_{1}^{k}}\sum_{a_{1}^{k-1}}P\left(X_{1}^{k}=a_{1}^{k-1},Y_{1}^{k}=b_{1}^{k}\right)\\ \; & \;\;\;\;\;\times \log P\left(Y_{k}=b_{k}\left|X_{1}^{k-1}=a_{1}^{k-1},Y_{1}^{k-1}=b_{1}^{k-1}\right.\right). \end{array}$$

Throughout this paper, “log” denotes the natural logarithm, and, by convention, we take $T\left(X_{1}\to Y_{1}\right):=H\left(Y_{1}\right)-H\left(Y_{1}\right)=0$.

Unlike mutual information, transfer entropy is, in general, asymmetric, i.e., $T\left(X_{1}^{k}\to Y_{1}^{k}\right)\neq T\left(Y_{1}^{k}\to X_{1}^{k}\right)$. The asymmetry of transfer entropy is characterized by the causally conditional entropy, which quantifies the entropy of Y conditioned on the causal part of X in addition to the history of Y. We say that X has no causal influence on Y when the causally conditional entropy is equal to the conditional entropy of Y. In this case, the transfer entropy is zero. Therefore, with this measure, we can quantify the strength and direction of the information flow between simultaneously observed systems.

Although transfer entropy is a measure widely used in neuroscience to quantify the amount of information that flows from one spike train to another, it only considers a finite block of states. In this sense, transfer entropy estimation is sensitive to faulty observations, which may lead to the identification of false causality. For a more comprehensive understanding of the system’s behavior, we may consider the estimation of an information flow rate. This idea leads to the following definition of the *transfer entropy rate*.

Since (X,Y) is a jointly stationary ergodic finite-alphabet process, we can define the *transfer entropy rate* from X to Y as

$$T\left(X\to Y\right)=\lim_{k\to \infty }T\left(X_{1}^{k}\to Y_{1}^{k}\right).$$

The existence of the limit can be checked as follows:

$$\begin{array}{cc} T(X\to Y) & =\lim_{k\to \infty }T\left(X_{1}^{k}\to Y_{1}^{k}\right)\\ \; & =\lim_{k\to \infty }\left(H\left(Y_{k}|Y_{1}^{k-1}\right)-H\left(Y_{k}|X_{1}^{k-1},Y_{1}^{k-1}\right)\right)\\ \; & =\lim_{k\to \infty }H\left(Y_{k}|Y_{1}^{k-1}\right)-\lim_{k\to \infty }H\left(Y_{1}|X_{1}^{k-1},Y_{1}^{k-1}\right)\\ \; & =H\left(Y_{0}|Y_{-\infty }^{-1}\right)-H\left(Y_{0}|X_{-\infty }^{-1},Y_{-\infty }^{-1}\right), \end{array}$$

where $H(Y_{0}|Y_{-\infty }^{-1})$ is the entropy rate H(Y) of the process Y and $H(Y_{0}|X_{-\infty }^{-1},Y_{-\infty }^{-1})$ is the causally conditional entropy rate H(Y|X). Thus,

T(X→Y)=H(Y)−H(Y|X).

The following proposition shows that, under appropriate conditions, the transfer entropy rate can be expressed in a simpler form.

**Proposition 1.**  *Suppose (X,Y) is a jointly stationary ergodic finite-alphabet variable-length Markov chain with memory no larger than k and with an arbitrary initial distribution. If, in addition, Y is also a variable-length Markov chain with memory no larger than k, then the transfer entropy rate T(X→Y) exists and it equals*

$$T\left(X\to Y\right)=I\left(Y_{0},X_{-k}^{-1}\left|Y_{-k}^{-1}\right.\right):=H\left(Y_{0}\left|Y_{-k}^{-1}\right.\right)-H\left(Y_{0}\left|X_{-k}^{-1},Y_{-k}^{-1}\right.\right).$$

**Proof.**  Since (X,Y) is a jointly stationary ergodic finite-alphabet process, we have the existence of T(X→Y) guaranteed. If (X,Y) is a variable-length Markov chain with memory no larger than *k* and with all positive transitions, then

$$\begin{array}{cc} H\left(Y_{0}\left|X_{-\infty }^{-1},Y_{-\infty }^{-1}\right.\right) & =\sum_{a_{-\infty }^{-1}}\sum_{b_{-\infty }^{0}}P\left(X_{-\infty }^{-1}=a_{-\infty }^{-1},Y_{-\infty }^{0}=b_{-\infty }^{0}\right)\\ \; & \;\;\;\;\;\times \log P\left(Y_{0}=b_{0}\left|X_{-\infty }^{-1}=a_{-\infty }^{-1},Y_{-\infty }^{-1}=b_{-\infty }^{-1}\right.\right)\\ \; & =\sum_{a_{-k}^{-1}}\sum_{b_{-k}^{-1}}P\left(X_{-k}^{-1}=a_{-k}^{-1},Y_{-k}^{0}=b_{-k}^{0}\right)\\ \; & \;\;\;\;\;\times \log P\left(Y_{0}=b_{0}\left|X_{-k}^{-1}=a_{-k}^{-1},Y_{-k}^{-1}=b_{-k}^{-1}\right.\right)\\ \; & =H\left(Y_{0}\left|X_{-k}^{-1},Y_{-k}^{-1}\right.\right). \end{array}$$

If, in addition, the process Y is itself a variable-length Markov chain with memory no larger than *k*, then, in a very similar way, we can show that the entropy rate H(Y) is simply $H\left(Y_{0}\left|Y_{-k}^{-1}\right.\right)$. Therefore,

$$T\left(X\to Y\right)=H\left(Y_{0}\left|Y_{-k}^{-1}\right.\right)-H\left(Y_{0}\left|X_{-k}^{-1},Y_{-k}^{-1}\right.\right)=I\left(Y_{0},X_{-k}^{-1}\left|Y_{-k}^{-1}\right.\right).$$

   □

Note that T(X→Y)=0 if and only if each $Y_{i}$, given its past $Y_{-\infty }^{i-1}$, is conditionally independent of $X_{-\infty }^{i-1}$. In other words, the transfer entropy rate is only zero in the absence of causal influence.

### 2.4. Transfer Entropy Rate Estimation

Since we generally do not have access to the probability distributions of the stationary processes whose possible causality relations are investigated, there are many methods to estimate the transfer entropy rate. This is particularly the case when recording neuronal and network signals, without or with equal external stimulation to the neurons, so that their activity is stationary, and inferring causal relationships, especially when dealing with different data formats. For a thorough review, we refer the reader to [20,46,47]. Thus, in this paper, we consider a *plug-in estimator* for the transfer entropy rate T(X→Y) between the jointly stationary ergodic chains X and Y (see Section 2.2).

Consider the positive integers *k* and *n* such that k≤n, and a given finite sample $\left(x_{-k+1}^{n},y_{-k+1}^{n}\right)\in {\left\{0,1\right\}}^{n+k}\times {\left\{0,1\right\}}^{n+k}$ from the jointly stationary ergodic chain (X,Y) with joint distribution P∈M. In this context, for any sequences $a_{0}^{k}\in {\left\{0,1\right\}}^{k+1}$ and $b_{0}^{k}\in {\left\{0,1\right\}}^{k+1}$, we define the *plug-in* estimate of *P* as

$${\hat{P}}_{n}^{\left(k\right)}\left(a_{0}^{k},b_{0}^{k}\right):=\frac{1}{n}\sum_{i=1}^{n}I\left\{{\tilde{x}}_{i-k}^{i}=a_{0}^{k},{\tilde{y}}_{i-k}^{i}=b_{0}^{k}\right\},$$

where I denotes the indicator function.

Note that ${\hat{P}}_{n}^{\left(k\right)}$ defines a probability measure on ${\left\{0,1\right\}}^{k+1}\times {\left\{0,1\right\}}^{k+1}$ induced by the sample $\left(X_{-k+1}^{n},Y_{-k+1}^{n}\right)$ from (X,Y). In this context, if $\left({\hat{X}}_{-k}^{0},{\hat{Y}}_{-k}^{0}\right)∼{\hat{P}}_{n}^{\left(k\right)}$, we may define the plug-in estimate for the transfer entropy rate $T\left(X\to Y\right)$ as

$${\hat{T}}_{n}^{\left(k\right)}\left(X\to Y\right):=T\left({\hat{X}}_{-k}^{0}\to {\hat{Y}}_{-k}^{0}\right).$$

Since (X,Y) is a jointly stationary ergodic chain with distribution P∈M, we have, by the ergodic theorem,

$$\lim_{n\to \infty }{\hat{P}}_{n}^{\left(k\right)}\left(a_{0}^{k},b_{0}^{k}\right)=P\left(X_{-k}^{0}=a_{0}^{k},Y_{-k}^{0}=b_{0}^{k}\right),\;P-a.s.,$$

for every positive integer *k* and $\left(a_{0}^{k},b_{0}^{k}\right)\in {\left\{0,1\right\}}^{k+1}\times {\left\{0,1\right\}}^{k+1}$. Thus, *P*-almost surely,

$$\lim_{k\to \infty }\lim_{n\to \infty }{\hat{T}}_{n}^{\left(k\right)}\left(X\to Y\right)=\lim_{k\to \infty }\lim_{n\to \infty }T\left({\hat{X}}_{-k}^{0}\to {\hat{Y}}_{-k}^{0}\right)=\lim_{k\to \infty }T\left(X_{-k}^{0}\to Y_{-k}^{0}\right)=T\left(X\to Y\right).$$

As discussed in Section III.2 in [48], p.174, we can take a single limit considering *k* as a function of *n*. If k(n)→+∞ whenever n→+∞ and $k\left(n\right)\leq \frac{\log n}{2}$, then the sequence {k(n):n≥1} is admissible to (X,Y) in the sense that

$$\lim_{n\to \infty }{\hat{P}}_{n}^{\left(k(n)\right)}\left(a_{0}^{k(n)},b_{0}^{k(n)}\right)=P\left(X_{-k(n)}^{0}=a_{0}^{k(n)},Y_{-k(n)}^{0}=b_{0}^{k(n)}\right),\;P-a.s..$$

Therefore, *P*-almost surely,

$$\lim_{n\to \infty }{\hat{T}}_{n}^{\left(k(n)\right)}\left(X\to Y\right)=\lim_{n\to \infty }T\left({\hat{X}}_{-k(n)}^{0}\to {\hat{Y}}_{k(n)}^{0}\right)=\lim_{n\to \infty }T\left(X_{-k(n)}^{0}\to Y_{-k(n)}^{0}\right)=T\left(X\to Y\right).$$

The asymptotic behavior of ${\hat{T}}_{n}^{\left(k\right)}\left(X\to Y\right)$ can also be described in terms of its probability distribution. According to [34], if X does not have a causal influence on Y, equivalently, if $T\left(X\to Y\right)=0$, then $2n{\hat{T}}_{n}\left(X\to Y\right)$ has an asymptotic $\chi^{2}\left(d\right)$ distribution, where the number of degrees of freedom *d* is equal to $2^{k}\left(2^{k}-1\right)$.

## 3. Hypothesis Test

Consider the problem of testing whether the binary time series generated by the process X has a causal influence on Y. In the present context, this corresponds to testing the null hypothesis that each random variable $Y_{i}$ is conditionally independent of $X_{i-k}^{i-1}$ given $Y_{i-k}^{i-1}$, within the larger hypothesis that the joint stationary and ergodic process (X,Y) is a variable-length Markov chain with order no larger than *k* and with all positive transitions.

Formally, each positive transition matrix $Q=Q_{\theta }$ for the process (X,Y) can be indexed by a parameter vector θ taking values in a $3\times 2^{k+1}$-dimensional open set Θ. The null hypothesis corresponding to each $Y_{i}$ being conditionally independent of $X_{i-k}^{i-1}$ given $Y_{i-k}^{i-1}$ is described by transition matrices $Q_{\theta }$ that can be expressed as

(4) $$Q_{\theta }\left(a_{0},b_{0}\left|a_{-k}^{-1},b_{-k}^{-1}\right.\right)=Q_{\theta }^{x}\left(a_{0}\left|a_{-k}^{-1},b_{-k}^{0}\right.\right)Q_{\theta }^{y}\left(b_{0}\left|b_{-1}^{-k}\right.\right),\;\left(a_{-k}^{0},b_{-k}^{0}\right)\in {\left\{0,1\right\}}^{k+1}\times {\left\{0,1\right\}}^{k+1}.$$

This collection of transition matrices can be indexed by parameters in a lower-dimensional parameter set Φ, which is an open subset of $R^{2^{k}\left(2^{k+1}+1\right)}$ and can be naturally embedded within Θ via a map h:Φ→Θ, with the property that all induced transition matrices $Q_{h(\phi )}$ satisfy the conditional independence property in (4).

To test the null hypothesis Φ within the general model Θ, we employ a likelihood test. The log-likelihood function $L_{n}\left(\theta \left|x_{-k+1}^{n},y_{-k+1}^{n}\right.\right)$ of θ given a sample $\left(x_{-k+1}^{n},y_{-k+1}^{n}\right)$ from the joint process (X,Y) can be expressed as

$$\begin{array}{cc} L_{n}\left(\theta \left|x_{-k+1}^{n},y_{-k+1}^{n}\right.\right) & =\log P_{\theta }\left(X_{1}^{n}=x_{1}^{n},Y_{1}^{n}=y_{1}^{n}\left|X_{-k+1}^{0}=x_{k+1}^{0},Y_{-k+1}^{0}=y_{-k+1}^{0}\right.\right)\\ \; & =\log\left(\prod_{i=1}^{n}Q_{\theta }\left(x_{i},y_{i}\left|x_{i-k}^{i-1},y_{i-k}^{i-1}\right.\right)\right) \end{array}$$

where $P_{\theta }$ denotes the law of (X,Y) with transition matrix $Q_{\theta }$. Then, the likelihood ratio test statistic is the difference

$$\Delta_{n}=2\left\{\max_{\theta \in \Theta }L_{n}\left(\theta \left|x_{-k+1}^{n},y_{-k+1}^{n}\right.\right)-\max_{\phi \in \Phi }L_{n}\left(h\left(\phi \right)\left|x_{-k+1}^{n},y_{-k+1}^{n}\right.\right)\right\}.$$

For our purposes, a key observation is that the statistic $\Delta_{n}$ is exactly equal to 2n times the plug-in estimator ${\hat{T}}_{n}^{\left(k\right)}\left(X\to Y\right)$.

**Proposition 2.**  *If (X,Y) is a variable-length Markov chain of memory no larger than k with all positive transition matrices Q on the finite alphabet {0,1}×{0,1} and with an arbitrary initial distribution, then*

$$\Delta_{n}=2n{\hat{T}}_{n}^{\left(k\right)}\left(X\to Y\right).$$

**Proof.**  The first maximum in the definition of $\Delta_{n}$ can be expressed as

$$\begin{array}{cc} \max_{\theta \in \Theta }L_{n}\left(\theta \left|x_{-k+1}^{n},y_{-k+1}^{n}\right.\right) & =\max_{\theta \in \Theta }\log\left(\prod_{i=1}^{n}Q_{\theta }\left(x_{i},y_{i}\left|x_{i-k}^{i-1},y_{i-k}^{i-1}\right.\right)\right)\\ \; & =\max_{Q}\sum_{i=1}^{n}\log Q\left(x_{i},y_{i}\left|x_{i-k}^{i-1},y_{i-k}^{i-1}\right.\right) \end{array}$$

where the last maximization is over all transition matrices *Q* with all positive entries. Thus,

$$\begin{array}{cc} \max_{\theta \in \Theta }L_{n}\left(\theta \left|x_{-k+1}^{n},y_{-k+1}^{n}\right.\right) & =\max_{Q}\sum_{a_{0}^{k}}\sum_{b_{0}^{k}}n{\hat{P}}_{n}^{\left(k\right)}\left(a_{0}^{k},b_{0}^{k}\right)\log Q\left(a_{0},b_{0}|a_{-k}^{-1},b_{-k}^{-1}\right)\\ \; & =-n\min\left\{\sum_{a_{0}^{k}}\sum_{b_{0}^{k}}{\hat{P}}_{n}^{\left(k\right)}\left(a_{0}^{k},b_{0}^{k}\right)\log\frac{{\hat{P}}_{n}^{\left(k\right)}\left(a_{k},b_{k}\left|a_{0}^{k-1},b_{0}^{k-1}\right.\right)}{Q\left(a_{k}b_{k}\left|a_{0}^{k-1},b_{0}^{k-1}\right.\right)}\right.\\ \; & \left.\;\;\;\;\;-\sum_{a_{0}^{k}}\sum_{b_{0}^{k}}{\hat{P}}_{n}^{\left(k\right)}\left(a_{0}^{k},b_{0}^{k}\right)\log{\hat{P}}_{n}^{\left(k\right)}\left(a_{k},b_{k}\left|a_{0}^{k-1},b_{0}^{k-1}\right.\right)\right\}. \end{array}$$

The above minimum is achieved by making

$$\sum_{a_{0}^{k}}\sum_{b_{0}^{k}}{\hat{P}}_{n}^{\left(k\right)}\left(a_{0}^{k},b_{0}^{k}\right)\log\frac{{\hat{P}}_{n}^{\left(k\right)}\left(a_{k},b_{k}\left|a_{0}^{k-1},b_{0}^{k-1}\right.\right)}{Q\left(a_{k}b_{k}\left|a_{0}^{k-1},b_{0}^{k-1}\right.\right)}=0.$$

Namely, when

$${\hat{P}}_{n}^{\left(k\right)}\left(a_{k},b_{k}\left|a_{0}^{k-1},b_{0}^{k-1}\right.\right)=Q\left(a_{k},b_{k}\left|a_{0}^{k-1},b_{0}^{k-1}\right.\right).$$

Therefore,

$$\max_{\theta \in \Theta }L_{n}\left(\theta \left|x_{-k+1}^{n},y_{-k+1}^{n}\right.\right)=n\left[H\left({\hat{X}}_{-k}^{-1},{\hat{Y}}_{-k}^{-1}\right)-H\left({\hat{X}}_{-k}^{0},{\hat{Y}}_{-k}^{0}\right)\right],$$

where $\left({\hat{X}}_{-k}^{0},{\hat{Y}}_{-k}^{0}\right)∼{\hat{P}}_{n}^{\left(k\right)}$.

The computation for the second maximum in the definition of $\Delta_{n}$ reduces to two different maximizations. Under the null hypothesis, *Q* admits the decomposition in (4), so that

$$\begin{array}{cc} \max_{\phi \in \Phi }L_{n}\left(h\left(\phi \right)\left|x_{-k+1}^{n},y_{-k+1}^{n}\right.\right) & =\max_{Q^{x}}\max_{Q^{y}}\sum_{i=1}^{n}\log\left(Q^{x}\left(x_{i}\left|x_{i-k}^{i-1},y_{i-k}^{i}\right.\right)Q^{y}\left(y_{i}\left|y_{i-k}^{i-1}\right.\right)\right)\\ \; & =\max_{Q^{x}}\sum_{i=1}^{n}\log Q^{x}\left(x_{i}\left|x_{i-k}^{i-1},y_{i-k}^{i}\right.\right)+\max_{Q^{y}}\sum_{i=1}^{n}\log Q^{y}\left(y_{i}\left|y_{i-k}^{i-1}\right.\right)\\ \; & =\max_{Q^{x}}\sum_{a_{0}^{k}}\sum_{b_{0}^{k}}{\hat{P}}_{n}^{\left(k\right)}\left(a_{0}^{k},b_{0}^{k}\right)\log Q^{x}\left(a_{k}\left|a_{0}^{k-1},b_{0}^{k}\right.\right)\\ \; & \;+\max_{Q^{y}}\sum_{b_{0}^{k}}{\hat{P}}_{n}^{\left(k\right)}\left(b_{0}^{k}\right)\log Q^{y}\left(b_{k}\left|b_{0}^{k-1}\right.\right)\\ \; & =n\left[-H\left({\hat{X}}_{-k}^{0},{\hat{Y}}_{-k}^{0}\right)+H\left({\hat{X}}_{-k}^{-1},{\hat{Y}}_{-k}^{0}\right)-H\left({\hat{Y}}_{-k}^{0}\right)+H\left({\hat{Y}}_{-k}^{-1}\right)\right]. \end{array}$$

Therefore, by the chain rule,

$$\Delta_{n}=2n\left[H\left({\hat{Y}}_{0}\left|{\hat{Y}}_{-k}^{-1}\right.\right)-H\left({\hat{Y}}_{0}\left|{\hat{X}}_{-k}^{-1},{\hat{Y}}_{-k}^{-1}\right.\right)\right]$$

which, recalling the definition of ${\hat{T}}_{n}^{\left(k\right)}\left(X\to Y\right)$, is precisely the claimed result.    □

As noted before, under the null hypothesis, that is, when $T\left(X\to Y\right)=0$, the distribution of $2n{\hat{T}}_{n}^{\left(k\right)}\left(X\to Y\right)$ is approximately $\chi^{2}$ with $2^{k}\left(2^{k}-1\right)$ degrees of freedom. Therefore, by Proposition 2, the likelihood ratio test statistic $\Delta_{n}$ is approximately $\chi^{2}$ distributed with $2^{k}\left(2^{k}-1\right)$ degrees of freedom. Note that this limiting distribution does not depend on the distribution of the underlying process (X,Y), except through the memory length *k*. Therefore, we can decide whether the data offer strong enough evidence to reject the null hypothesis by examining the value of $\Delta_{n}$. Given a threshold α∈(0,1), if $\delta_{n}$ is the observed value of $\Delta_{n}$ and $P(\Delta_{n}>\delta_{n})\leq \alpha$, then the causality hypothesis can be rejected at the significance level α. The algorithm for conducting this hypothesis test is described below in Algorithm 1.
**Algorithm 1** Causal influence test.**Input:** Data $\left(x_{-k+1}^{n},y_{-k+1}^{n}\right)\in {\left\{0,1\right\}}^{n+k}\times {\left\{0,1\right\}}^{n+k}$;
      Significance level α∈(0,1);
      Test statistic $\Delta_{n}$.
**Output:** Decision: Reject or not reject the causality hypothesis $H_{0}$.
  $\delta_{n}\leftarrow \Delta_{n}\left(x_{-k+1}^{n},y_{-k+1}^{n}\right)$.
  $p\leftarrow P(Q>\delta_{n})$, where $Q∼\chi_{2^{k}\left(2^{k}-1\right)}^{2}$.
  **if** 
p≤α 
**then**
     Reject $H_{0}$;
  **else if** 
p>α 
**then**
     Not reject $H_{0}$.
  **end if**

**Figure 1:**
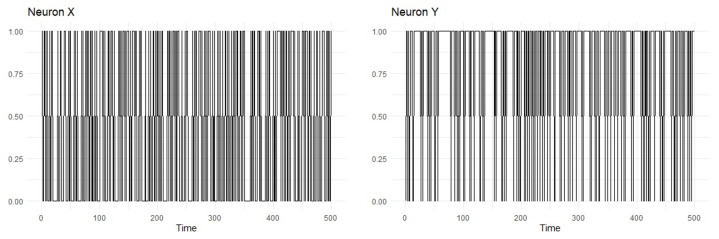
First five hundred observations of the time series X and Y generated from the neuronal network model described in Section 2, with memory no larger than k=3 and synaptic weights $\omega_{x\to y}=10$ and $\omega_{y\to x}=0$.

**Example 1.**  *Consider a sample $\left(x_{-k+1}^{n},y_{-k+1}^{n}\right)\in {\left\{0,1\right\}}^{n-k}\times {\left\{0,1\right\}}^{n-k}$ of length n=40,000 generated from a microcircuit composed of two neurons whose activities are modeled as in the neuronal network model described in Section 2. In this case, we consider the jointly stationary ergodic variable-length Markov chain (X,Y) with memory no larger than k=3. In this microcircuit, there is a strong excitatory connection from neuron X to neuron Y but no connection from neuron Y to neuron X, i.e., $\omega_{x\to y}=10$ and $\omega_{y\to x}=0$. In Figure 1, we illustrate the signals from the neurons generated by the neural model for this parameter specification.*

*We conduct two hypothesis tests. In the first one, we are interested in testing the following hypotheses: $H_{0}:\omega_{x\to y}=0$ vs. $H_{1}:\omega_{x\to y}\neq 0$. In this case, the observed value of the test statistic is $\delta_{n}=5881.70$. Therefore, by setting a significance level of α=5%, we obtain p≈0. Hence, since p<α, we reject the null hypothesis. On the other hand, in the second test, the hypotheses are as follows: $H_{0}:\omega_{y\to x}=0$ vs. $H_{1}:\omega_{y\to x}\neq 0$. In this case, the observed value of the test statistic is $\delta_{n}=51.08$. Thus, by setting a significance level of α=5%, we obtain p=0.6612. Therefore, since p>α, we do not reject the null hypothesis.*

*A more comprehensive simulation study is given in the next section.*

## 4. Results on Simulated Data

A natural interest of this work is the application of transfer entropy in the study of effective connectivity between a pair of stochastic neurons. For this, we use synthetic data generated from the neuronal network model described in Section 2. To test the statistical significance of a connectivity value, we use the hypothesis test described in Section 3.

For the experiment conducted in this section, we consider two stochastic neurons with variable-length memory whose dynamics are given by the model introduced in Section 2. In this case, the neuronal network is a microcircuit composed of two neurons whose activities are modeled by the jointly stationary ergodic variable-length Markov chain (X,Y) with memory no larger than k=3. We select scenarios where the synaptic weights are either strong or weak. Based on different choices of these synaptic weights, we define the following four distinct cases:

- **Scenario 1:** There is a strong excitatory connection from neuron X to neuron Y but no connection from neuron Y to neuron X, i.e., $\omega_{x\to y}=10$ and $\omega_{y\to x}=0$. In this case, when Y is the postsynaptic neuron, we observe, on average, a firing proportion of 80%.
- **Scenario 2:** There is a weak excitatory connection from neuron X to neuron Y but no connection from neuron Y to neuron X, i.e., $\omega_{x\to y}=0.375$ and $\omega_{y\to x}=0$. In this case, when Y is the postsynaptic neuron, we observe, on average, a firing proportion of 52%.
- **Scenario 3:** There is a weak inhibitory connection from neuron X to neuron Y but no connection from neuron Y to neuron X, i.e., $\omega_{x\to y}=-0.375$ and $\omega_{y\to x}=0$. In this case, when Y is the postsynaptic neuron, we observe, on average, a firing proportion of 48%.
- **Scenario 4:** There is a strong inhibitory connection from neuron X to neuron Y but no connection from neuron Y to neuron X, i.e., $\omega_{x\to y}=-10$ and $\omega_{y\to x}=0$. In this case, when Y is the postsynaptic neuron, we observe, on average, a firing proportion of 30%.

In all scenarios, when X is the postsynaptic neuron, we observe, on average, a firing proportion of 50%. In addition, for each scenario, we consider four different sample sizes: n=5000; *n* = 10,000; *n* = 20,000; and *n* = 40,000, representing *n* bins of 10 ms. These are typical recording times in electrophysiological experiments. Note that with these choices of sample sizes, we have $k<\frac{\log n}{2}$, for all *n*, which ensures the convergence results of the transfer entropy rate estimator discussed in Section 2.4. For each scenario and sample size, 100 replicates are generated, and the test is conducted on each of them.

**Figure 2:**
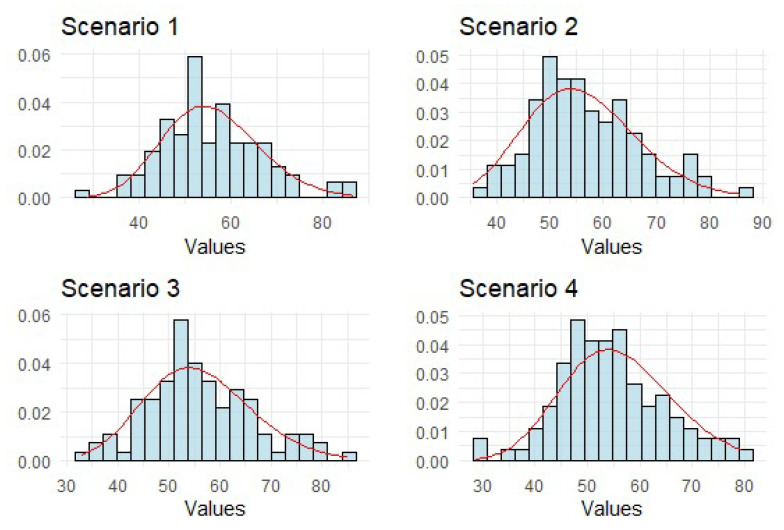
Histogram approximation of the distribution of statistics $\Delta_{n}$, based on 100 repetitions of the test on samples of length *n* = 400,000. The red curve shows the density of the theoretically predicted limiting $\chi^{2}\left(56\right)$ distribution.

Using 100 repetitions for the test on samples of length *n* = 40,000, the empirical distribution of the statistic $\Delta_{n}$ is estimated, as shown in Figure 2, to be in close agreement with the theoretically predicted $\chi^{2}\left(56\right)$ limiting distribution in all scenarios.

In Table 1, we show the fraction of times, out of 100 simulations of the neuronal network model, that the test rejects the null hypothesis of the absence of causal influence for four different significance levels: α = 0.1%, α=1%, α=5%, and α=10%. We can observe that, as expected (and desired), in scenarios 1 and 2, where there is a strong connection from neuron X to neuron Y (excitatory with $\omega_{x\to y}=10$ and inhibitory with $\omega_{x\to y}=-10$, respectively), the test detects the connection in 100% of the cases regardless of the sample size and significance level. However, in scenarios 2 and 3, where there is a weak connection (excitatory with $\omega_{x\to y}=0.375$ and inhibitory with $\omega_{x\to y}=-0.375$, respectively), the test struggles to detect the connection with small sample sizes and low significance levels, and its performance improves as we increase the sample size and the significance level. Furthermore, in all scenarios, the test does not reject the null conditional independence hypothesis when Y is the presynaptic neuron and X is the postsynaptic neuron. In fact, there is no connection from neuron Y to neuron X$\left(\omega_{y\to x}=0\right)$. Therefore, the results are in agreement with the nature of the data.

**Figure 3:**
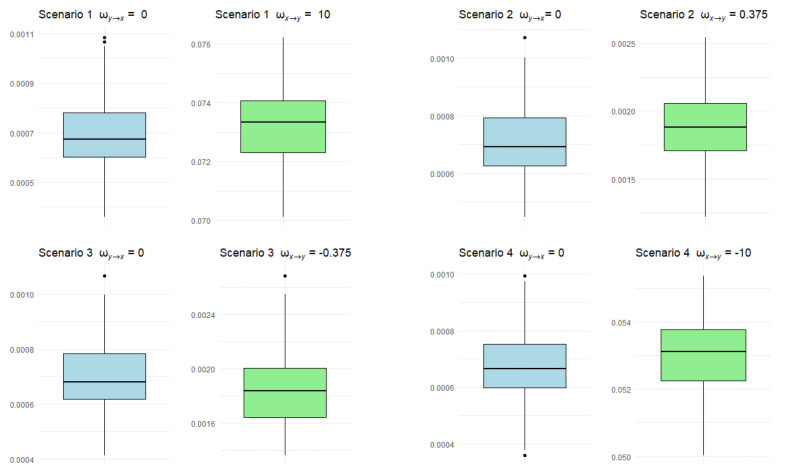
Box plots of the estimated transfer entropy rates for each of the 100 samples of length *n* = 40,000 generated by the neuronal network model.

In Figure 3, we display the distributions of the estimated transfer entropy rates for each of the 100 samples of length *n* = 40,000 generated by the neuronal model using box plots. We can observe that in scenarios 1 and 4, where there is a strong connection from neuron X to neuron Y, the estimated values are similar and greater than those estimated in scenarios 2 and 3, where there is a weak connection from neuron X to neuron Y. On the other hand, in all scenarios, there is no connection from neuron Y to neuron X, and the estimated values tend to be lower than those obtained in the aforementioned situations.

## 5. Conclusions

In this work, we studied the effective connectivity between neurons through a hypothesis test whose test statistic is based on the plug-in estimator of the transfer entropy rate. Effective connectivity refers to the causal interactions among distinct neural units, whereas anatomical connectivity and functional connectivity refer to anatomical links or loosely defined statistical dependencies between units, respectively [49]. Our work demonstrates how to properly use transfer entropy to measure the flow of information between sequences and explore its use in determining effective neuronal connectivity.

Understanding effective connectivity has long been a central challenge in neuroscience [1,50,51,52]. The identification of connectivity has garnered significant interest in recent years, primarily due to advancements enabling the simultaneous recording of a vast number of neurons [53,54,55,56]. Essentially, we now exploit the understanding that synaptic connections induce voltage fluctuations capable of triggering postsynaptic action potentials. These subtle effects modulate spike timing within a spike train, discernible under specific conditions. By iteratively applying inference techniques to extensive datasets, crucial connectivity maps for understanding the brain are generated. A successful recent example is the inference of the small central pattern-generating circuit in the stomatogastric ganglion of the crab *Cancer borealis* [57]. This circuit is known and so it is amenable to this type of analysis. Yet, it is a challenging circuit because pharmacological manipulations alter the neuronal intrinsic dynamics and synaptic communication, as clearly shown by the authors. However, for the majority of other living systems, challenges persist in this process, including the stochastic nature of neurons originating from a highly random environment, leading to confounding factors that are challenging to disambiguate, as well as the selection of an appropriate and refined metric capable of addressing such issues.

Our analysis indicates that the hypothesis testing framework described in this paper can be a useful exploratory tool for providing conclusive biologically relevant findings. Here, we showed that this test reliably detected effective connectivity when two signals are generated from a neuronal network model in which neurons are stochastic with variable-length memory.

A second contribution of this work concerns the relationship between the synaptic weight values set for the neuronal network model described in Section 2 and the estimates of the transfer entropy rate (see Figure 3). We observed that synaptic weight values close to zero lead to estimates that are also close to zero. On the other hand, the farther from zero the synaptic weight values, the larger the estimates of the transfer entropy rate. Therefore, empirical transfer entropy effectively translates the types of connections existing between the neurons in the network.

One avenue for future research stemming from this work is the testing and validation of our studies with experimental data. Difficulties may arise, given that not many circuits are completely known to act as ground-truth data, with some exceptions such as *Cancer borealis* [57] or *C. elegans* [58]. An intermediate step would, therefore, involve applying simulations with biophysically grounded neurons based on the Hodgkin–Huxley model [59]. The inclusion of a stochastic background at different levels of the model (ion channels, synapses, network) would highlight the advantages of our approach and allow for further extensions.

The findings of this study suggest that the method is both robust and versatile, accurately deducing effective connectivity among neurons possessing various synaptic characteristics. We utilize synaptic values aligned with both strong and weak neuronal connections in the brain. Regarding its versatility, this implies that the technique can be enhanced and extended to more intricate systems, considering the influence of confounding factors such as stimuli or additional spike trains from third parties. 

## Figures and Tables

**Table 1:** Fraction of times, out of 100 simulations of the neuronal network model, that the test rejects the null hypothesis of the absence of causal influence for four different significance levels and sample sizes.

$H_{0}:T\left(X\to Y\right)=0$ vs. $H_{1}:T\left(X\to Y\right)>0$						$H_{0}:T\left(Y\to X\right)=0$ vs. $H_{1}:T\left(Y\to X\right)>0$				

**SCENARIO 1** $\;\omega_{x\to y}=10$						**SCENARIO 1** $\;\omega_{y\to x}=0$				
	α=0.1%	α=1%	α=5%	α=10%			α=0.1%	α=1%	α=5%	α=10%
n=5000	100	100	100	100		n=5000	0	0	1	4
n=10,000	100	100	100	100		n=10,000	0	0	0	2
n=20,000	100	100	100	100		n=20,000	0	1	3	4
n=40,000	100	100	100	100		n=40,000	0	1	1	3

**SCENARIO 2** $\;\omega_{x\to y}=0.375$						**SCENARIO 2** $\;\omega_{y\to x}=0$				
	α=0.1%	α=1%	α=5%	α=10%			α=0.1%	α=1%	α=5%	α=10%
n=5000	5	19	36	45		n=5000	0	0	4	8
n=10,000	18	44	66	76		n=10,000	0	1	4	10
n=20,000	71	88	95	99		n=20,000	0	0	2	8
n=40,000	100	100	100	100		n=40,000	0	1	8	10

**SCENARIO 3** $\;\omega_{x\to y}=-0.375$						**SCENARIO 3** $\;\omega_{y\to x}=0$				
	α=0.1%	α=1%	α=5%	α=10%			α=0.1%	α=1%	α=5%	α=10%
n=5000	2	12	23	42		n=5000	0	1	3	6
n=10,000	13	27	58	72		n=10,000	0	1	9	13
n=20,000	65	85	95	95		n=20,000	0	1	5	8
n=40,000	100	100	100	100		n=40,000	0	1	7	10

**SCENARIO 4** $\;\omega_{x\to y}=-10$						**SCENARIO 4** $\;\omega_{y\to x}=0$				
	α=0.1%	α=1%	α=5%	α=10%			α=0.1%	α=1%	α=5%	α=10%
n=5000	100	100	100	100		n=5000	0	1	4	10
n=10,000	100	100	100	100		n=10,000	0	1	3	4
n=20,000	100	100	100	100		n=20,000	0	0	5	10
n=40,000	100	100	100	100		n=40,000	0	0	4	8

## Data Availability

Numerical simulations and data are freely available at https://github.com/Joao-Izzi/IC_codes (accessed on 1 April 2024).
